# New Algorithm to Determine True Colocalization in Combination with Image Restoration and Time-Lapse Confocal Microscopy to Map Kinases in Mitochondria

**DOI:** 10.1371/journal.pone.0019031

**Published:** 2011-04-29

**Authors:** Jorge Ignacio Villalta, Soledad Galli, María Florencia Iacaruso, Valeria Gabriela Antico Arciuch, Juan José Poderoso, Elizabeth Andrea Jares-Erijman, Lía Isabel Pietrasanta

**Affiliations:** 1 Centro de Microscopías Avanzadas, Facultad de Ciencias Exactas y Naturales, Universidad de Buenos Aires, Buenos Aires, Argentina; 2 Departamento de Química Orgánica, Facultad de Ciencias Exactas y Naturales, Universidad de Buenos Aires, CIHIDECAR, CONICET, Buenos Aires, Argentina; 3 Consejo Nacional de Investigaciones Científicas y Técnicas, Buenos Aires, Argentina; 4 Laboratory of Oxygen Metabolism, University Hospital “José de San Martín,” Facultad de Medicina, Universidad de Buenos Aires, Buenos Aires, Argentina; Texas A&M University, United States of America

## Abstract

The subcellular localization and physiological functions of biomolecules are closely related and thus it is crucial to precisely determine the distribution of different molecules inside the intracellular structures. This is frequently accomplished by fluorescence microscopy with well-characterized markers and posterior evaluation of the signal colocalization. Rigorous study of colocalization requires statistical analysis of the data, albeit yet no single technique has been established as a standard method. Indeed, the few methods currently available are only accurate in images with particular characteristics. Here, we introduce a new algorithm to automatically obtain the true colocalization between images that is suitable for a wide variety of biological situations. To proceed, the algorithm contemplates the individual contribution of each pixel's fluorescence intensity in a pair of images to the overall Pearsońs correlation and Manders' overlap coefficients. The accuracy and reliability of the algorithm was validated on both simulated and real images that reflected the characteristics of a range of biological samples. We used this algorithm in combination with image restoration by deconvolution and time-lapse confocal microscopy to address the localization of MEK1 in the mitochondria of different cell lines. Appraising the previously described behavior of Akt1 corroborated the reliability of the combined use of these techniques. Together, the present work provides a novel statistical approach to accurately and reliably determine the colocalization in a variety of biological images.

## Introduction

The specific localization of biomolecules in the subcellular compartments is extremely important as a variety of functions may be derived. This is usually studied by fluorescence microscopy and posterior evaluation of the colocalization of the molecule of interest in a definite region in the cell. Most frequently, image processing for colocalization is visual-based and therefore highly prone to bias. In the last years, a variety of methods have been described to analyze colocalization quantitatively in different biological systems, based on statistic analysis of pixel intensity distributions and/or object recognition [1]–[6] and have been integrated in different software, some of which are freely accessible. However, these methods are often applicable only in images with certain characteristics [3]–[6] or relay further on visual estimation [2], [7]. Indeed, in the last year two improved methods have been developed. Fletcher and coworkers [5] established a new method based on Monte Carlo randomization that works with voxel-based, intensity-based, object-based, and nearest-neighbour metrics to measure the statistical significance of colocalization. Seemingly, Ramírez and co-workers [6] introduced a confined displacement algorithm based on image correlation spectroscopy in combination with Manders colocalization coefficients to quantify true and random colocalization of a given florescence pattern. However, these two methods assume that the image can be divided into isolated ‘dots’ or ‘blobs’ and therefore that each object can be isolated from the other, which depends on the data being punctate in nature. This is characteristic of high frequency data such as the immunofluorescence of membrane proteins, but not of low frequency data such as calcium concentration measurements, or of molecules that are widely dispersed throughout the cytosol [5]. Indeed, a major challenge in quantitative colocalization has been to specifically determine the localization of widely distributed proteins for example in mitochondria or Golgi, due to the small size of these organelles. In a different approach, Costes and coworkers [3] proposed an automated method for segmentation, the calculation of modified Manders coefficients and a statistical validation of colocalization by a combination of block scrambling and probability density functions [3]. Unfortunately, this method is suitable only for scenarios with very low and symmetric signal densities, which is usually not the case in biological samples [6], [8]. Here, we introduce a new algorithm to automatically detect the true colocalization in a pair of images that overcomes the limitations of previous methods. The algorithm contemplates the individual fluorescence intensity contribution of every pixel in a pair of images to the Pearson's correlation and Manders' overlap coefficient. The accuracy and reliability of the algorithm was corroborated in a variety of simulated and real images that contemplated the characteristics of real biological data. The use of this algorithm together with the construction of the more informative maps derived from the colocalization coefficients allowed us to directly observe in which parts inside the cell the colocalization was particularly relevant.

An additional task to overcome in the analysis of colocalization is the limit in the resolution imposed by the optical system. Image deconvolution is a useful tool to more unequivocally address the localization of a molecule in a small compartment as this procedure eliminates the contribution of optical diffraction and therefore restores the “real” image from the acquired samples [9]. We combined the use of the algorithm with image restoration by deconvolution and time-lapse confocal microscopy to gain insights into the localization and trafficking of MEK into the mitochondria of different cellular models. The MEKs (ERK1/2 kinases) function within signaling cascades that regulate a variety of cellular processes including proliferation, differentiation and development, among others [10]. The MEKs constitute an evolutionary conserved group of dual specificity protein kinases that includes three highly homologous mammalian isoforms: MEK1a (44 kDa), MEK1b (41 kDa) and MEK2 (45 kDa) [11]–[12]. MEKs phosphorylate with high specificity both regulatory Thr and Tyr residues of ERKs [13]. Specificity is achieved by conserved docking domains present in both kinases [14]. MEKs were initially reported to localize primarily in the cytosol, while subsequent evidence suggested that MEK1 was able to shuttle into the nucleus, but was rapidly exported by a nuclear export signal [15]–[17]. Recent evidence suggests the mitochondria as an intermediate regulatory station between cytosol and nuclei [18]–[22]. Indeed, we observed that ERK translocation into or out of the organelle was regulated by oxidative stimuli or serum, and that the formation of MEK-ERK complexes in these organelles was regulated by the oxidation of a redox sensitive cysteine in ERK2 and the impeded binding to MEK caused ERK2 retention in the organelle in detriment of its shuttle into the nuclei [19]. In a similar approach, Antico-Arciuch and co-workers confirmed the presence of Akt1 in mitochondria of NIH cells and that mitochondrial redox status regulated Akt1 progression to the nuclei [22]. Indeed, mitochondria regulate Akt1 traffic through posttranslational modifications; phosphorylation of Akt Thr308 occurs in the organelle and mitochondrial derived H_2_O_2_ affects Akt-PDK interaction by the selective oxidation of Akt1 Cys^310^ to sulfenic or cysteic acid [22]. Although we showed that the traffic of ERK to mitochondria is regulated, i.e., by serum or H_2_O_2_ [19]–[20], little is known about the biological relevance of the localization of its activator MEK to mitochondria or the regulation of MEK translocation into and out of the organelle. Combining the use of the new colocalization algorithm with image restoration and time-lapse confocal microscopy, we demonstrate the presence of MEK in mitochondria of HeLa and MEF cells and that MEK localization in the organelle is favored in proliferative conditions.

Together, the present work provides a novel statistical approach to precisely and consistently ascertain the colocalization in a series of biological images. The algorithm, written in Matlab code, was integrated in a graphical users interface for easy accessibility and is freely available as a source (www.mathworks.com/matlabcentral/fileexchange/30665-villalta-et-al-s-colocalization-algorithm).

## Materials and Methods

### Cell culture

Human cervical carcinoma HeLa cells were maintained in Dulbecco's modified Eagle's medium (D-MEM) nutrient mixture F-12 HAM with 10% fetal calf serum (FCS) and 80 µg/ml gentamycin at 37°C in 5% CO_2_. NIH/3T3 cells were grown in DMEM F-12 with 10% calf serum plus gentamycin as above. Mouse embryonic fibroblast (MEF) *erk1−/−* cells (kind gift of Dr. Jacques Pouyssegur to Dr. Thomas Jovin lab) were cultured in D-MEM plus GlutaMAX™-1 (Gibco) with 10% FCS, streptomycin and penicillin, supplemented with MEM non-essential amino acids (Gibco) as above. The medium was replaced twice per week. Passages were made by trypsinization of confluent monolayers. PC12 cells were cultured and differentiated as described elsewhere [23]. Mitochondria were purified from cell lines as previously described [20].

### Plasmids

hERK1-GFP was a gift of Diane Lidke [24]. DsRed-MEK was the kind gift of Phillippe Stork [25]. The cloning of Akt1-GFP and its phosphorylation mutants and hERK1-Dronpa was described elsewhere [20], [22]. p75-YFP was the kind gift of Francisca Bronfman (Facultad de Ciencias Biológicas Pontifica Universidad Católica de Chile). The cloning of TrkA-CFP is described elsewhere (Iacaruso *et al*., in preparation). Mito-RFP and mito-YFP were from Clontech.

### Fluorescence labeling

Cells were grown on Lab-Tek Chambered Borosilicate Coverglass System (Nunc) for *in vivo* experiments or on coverslides when cells were further fixed and immuno-stained. Cell transfection was accomplished using Lipofectamine 2000 (Invitrogen) according to manufacturer's instructions. When appropriate, cells were stained with specific mitochondrial markers, MitoTracker Green, MitoTracker RED CMXRos or MitoTracker DeepRed (Invitrogen, 100 nM, 45 min at 37°C). For fixation and labelling cells were incubated in 4% paraformaldehyde 10 min at room temperature, blocked and permeabilized in PBS, 0.05% Triton X-100, 0.1% BSA, and labelled with Phalloidin-FITC and Phalloidin-Rodamine in the same buffer 1 h at room temperature.

### Confocal microscopy

Images were acquired in an Olympus FV1000 spectral confocal microscope (Olympus, Latin America) with a 60×1.35 NA oil immersion objective. Excitation and filters were as follows: CFP, 458 nm excitation, emission 470–510 nm; GFP, YFP-mito and FITC, 488 nm excitation, emission BP 500–530 nm; p75-YFP, 515 nm excitation, emission 530–630 nm; RFP, MitoTracker RED CMXRos and Rodhamine, 543 nm excitation, emission BP 555–655 nm; MitoTracker DeepRed, 635 nm excitation, emission 650–750 nm. Images were acquired in a sequential mode. Bleed-through was checked by imaging of samples labeled with a single fluorophore and acquiring dual channel images with the same setup used for the co-labeled system. We detected and corrected channel cross-talk between YFP and DsRed. There was no registration shift between images.

For time-lapse imaging, cells were stimulated with 10% FCS or 50 µm H_2_O_2_ and imaged every 1–2 min over a period of 20 min at 37°C. Three-8 equidistant (0.5 µm) planes were evaluated for each cell (field). Images were scanned as 137×137 or 109×109 nm per pixel.

For image deconvolution stacks of 30–40 equidistant (0.1 µm) planes were evaluated. Images were scanned as 66×66 or 55×55 nm per pixel.

### Image analysis

The image and statistical analysis of colocalization and translocation kinetics was performed with Matlab (MathWorks, Natick, MA) and DIPimage (image processing toolbox for Matlab, Delft University of Technology, The Netherlands). Background levels were obtained by measuring the mean intensity of each signal outside the cells and were subtracted; negative pixel values were clipped to zero. Colocalization indexes *m1* and *m2*, Manders Overlap coefficient (*R*) and Pearsońs correlation coefficient (*r*) were calculated as previously described (Table 1) [1]–[2], [26]. Colocalization was determined with our new algorithm, described below. For the analysis of time-lapse images, either we determined a colocalization mask or, comparatively, we delimited a mitochondrial compartment when the MitoTracker fluorescence intensity was twice above the mean of the whole image. The cellular compartment was delimited when GFP (for Akt) or DsRed (for MEK) fluorescence intensity was over the mean fluorescence intensity of the whole image. Akt-GFP or DsRed-MEK kinetics were studied only in mitochondria of transfected cells, and thus a new mask was determined by the combination of both the cellular mask and the MitoTracker generated mask when we delimited the compartments manually. The change in GFP and DsRed fluorescence intensity was evaluated inside mitochondria and normalized to the whole cell fluorescence intensity to contemplate photobleaching.

**Table 1:** Colocalization coefficients.

Correlation Coefficient	Formula	Equation
Pearsońs correlation coefficient (*r*)	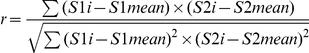	*Eq. 1*
Manders Overlap Coefficient (*R*)	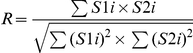	*Eq. 2*
*m1* and *m2*	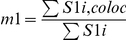 ; 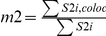	*Eq. 3; Eq. 4*
*k1* and *k2*	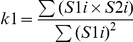 ; 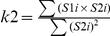	*Eq. 5; Eq. 6*

S1*i* represents signal intensity of pixels in the channel 1 and S2*i* represents signal intensity of pixels in the channel 2; S1*mean* and S2*mean* reflect the average intensities of these respective channels. S1*i*,*coloc* and S2*i*,*coloc* are those pixels that also display non-random fluorescence in the other channel.

Image deconvolution was performed with Huygens Deconvolution Software (Scientific Volume Imaging). The point-spread function (PSF) was calculated theoretically based on the imaging parameters. Signal-to-noise ratio was set to 30–40. Deconvolution was performed by the Maximum Likelihood Estimation (MLE) algorithm. Background was subtracted automatically.

The maps for the coefficients were computed by estimating the contribution of each single pixel to the coefficient. Thus, single pixel value corresponds to the value they generate according to the equations in Table 1, divided by the whole equation denominator.

### New colocalization algorithm

Starting with the whole population of pixels of the green and red channel images, the algorithm constructs the *r* map and the *R* map, and then calculates the product of the *r*×*R* map, which we call mixed map. Subsequently, it selects pixels that contribute highly positively (colocalizing) or highly negatively (anti-colocalizing) to *r*×*R* -this is, those pixels that contribute to the product of numerators above or below certain thresholds- and classifies them into two different groups. The thresholds were arbitrarily set as the 80% of the maximum value of the mixed map for colocalizing pixels and the 80% of the minimum value of the mixed map for anti-colocalizing pixels. With the remaining population of pixels the algorithm constructs a new mixed map and again classifies and adds these pixels into one of the former groups. The algorithm continues with this procedure until more than 1% of the pixels classified in one step belong to the background –defined as zero intensity- of any of the channels. At the end of this procedure several masks are achieved –one per round of classification- with either the colocalized or anti-colocalized pixels.

In the next step, the algorithm determines which of all the colocalized pixels masks is the most appropriate. With this aim, the algorithm computes the *R* for the pixels enclosed by every colocalizing mask, thus it achieves one *R* value per round of classification. The minimum *R* is subtracted from all the *R* values and then plotted vs the classification round (*R*-*R*_min_ vs. round of classification). Subsequently, the algorithm calculates the area under the former function and determines the most appropriate colocalizing mask that one attained in the round where the 86% of the area is reached. This value was empirically determined by running the algorithm on different simulated image sets (different object densities) and different colocalization extents (for details see Text S1 and Fig. S1). We chose 86% of the area as this value rendered the best approximation to the true colocalization and the minimum detection of false positives.

### Test of significance for the Manders Overlap coefficient (*R*)

In the same way Costes and coworkers derived a statistical significance test to evaluate the significant correlation in a pair of images [3], we derived a statistical significance test to evaluate the probability (*P*) that the measured value of *R* from the two images is significantly greater than values of *R* that would be calculated if there was only random overlap. Briefly, by repeatedly scrambling the pixels in one image, and then estimating *R* of this image with the other (unscrambled) image we generate the empirical probability distribution of the amounts of random overlap specifically for these two images. By comparing the *R* measured for the original unscrambled image with this distribution we determine whether significant colocalization exists for a predefined probability for significance (Students *t* distribution).

Since single pixel intensity is correlated with its neighboring pixels due to optical limitations, i.e., the PSF, for randomization we divided the images into independent blocks of approximately the size of the PSF, when necessary. We then scrambled the blocks instead of the individual pixels. We performed 20 randomizations and chose a *P* value of 0.95% to indicate significant (true) colocalization.

### Simulated images

We generated 160×160 pixels images with a determined number of pixel-sized objects with a range intensity distribution of 30–155 for both channels. Colocalized pixels had identical non-zero intensities. The density of each channel varied and ranged from 5% to 30% for each green or red objects (i.e., 5% to 30% of the area is covered by green objects; the same for red objects). Simulations covering the full range of possible colocalization were performed, from all green or red objects colocalized to no colocalization. Noise with intensities distributed uniformly between 10 and 30 was added to both channels.

Alternatively, we generated 256×256 pixels images. Objects consisted of 220 pixels circles with random intensities from 40 to 160. We deliberately set 20 objects as colocalized. We added 15 objects to each image randomly, and this increased the percentage of colocalized signal, as some of the balls partially overlapped. The colocalization achieved was 7%. Finally, normally distributed noise (mean  = 10, Standard deviation  = 15) was added to the whole image.

## Results

### New colocalization algorithm, rationale

In accord to *r* (Table 1), two images are correlated if *r*>0, independently distributed if *r*∼0 or negatively correlated if *r*<0. It is generally assumed that there is colocalization between two images if the overall *r* is positive. As *r* is invariant to background and intensity scales, it provides a robust estimator for colocalization [1]. However, the major drawback of this estimator is its lack of biological meaning and the fact that in a variety of cases, the ratio colocalized/total image area is so small that the overall estimator remains around 0 or slightly positive [27]. Indeed, the fact that the overall *r* is either 0 or even negative does not imply the complete absence of colocalized signal, rather than the null or negatively contributing pixels are abundant (Fig. S2A). Manders coefficients *m1* and *m2* are more biologically meaningful (Table 1) [2] as they contemplate the colocalization area between the images independently of their correlation. Their major disadvantage is that they relay further on visual estimation to establish the cut-off to determine signal from noise, or more complex, random from specific localization.

To develop this algorithm we assumed that in a dual-channel image, those pixels that contributed positively to *r* accounted for the colocalized population of pixels, while those pixels that contributed negatively to *r* corresponded to the mutually exclusive (anti-colocalized) population of pixels. Depending on the proportion of these populations the overall *r* may be positive, negative or null (Fig. S2A). We first studied the behavior of *r* and *R*. For this, we plotted the contribution to *r* and *R* of each potential green-red fluorescence intensity combination for a pair of images with fluorescence ranging from 0–255 with a mean of 75 (Fig. S2B). There are different fluorescence intensity combinations that contribute to *r* or *R* with the same value and these constitute the coefficient's level curves (Fig. S2B, highlighted in colors). In other words, level curves indicate those green and red fluorescence intensity pixels that contribute equally to *r* or *R*. In addition, pixels can contribute negatively to *r* numerator, and this is achieved when either the green or red channel fluorescence is below its respective mean fluorescence intensity; *R* numerator cannot attain negative values (Fig. S2B, left panels). The same is true for images with other fluorescence intensity maxima and mean.

**Figure 1:**
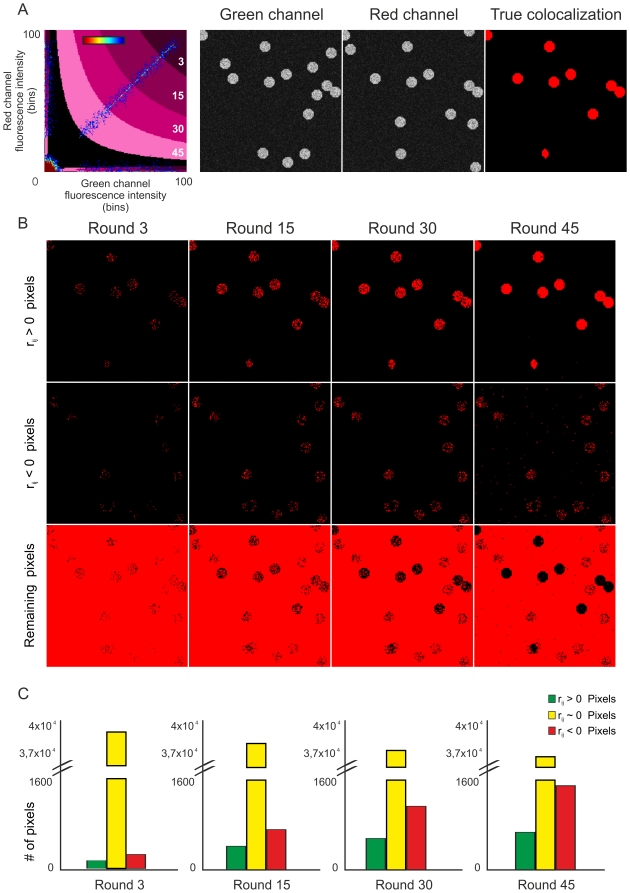
New colocalization algorithm, description. A) Two-dimensional fluorescence histogram (left panel) of the images on the right. The pixel distribution is highlighted in jet colour map. Pixels displaying fluorescence only in the green or red channel lie on the *x* or *y* axis, respectively, pixels displaying fluorescence in both channels are placed along the diagonal, pixels with background signal are accumulated at the origin of the histogram. Four representative rounds of pixel classification are highlighted in pink colours (rounds 3, 15, 30 and 45). On the right, 256×256 pixel sized images with 15 “green channel” and 14 “red channel” objects (circles) with random intensities varying between 40 and 160. A definite number of objects were deliberately set as colocalized (True colocalization). Jet colour bar, number of pixels. Bins, intervals of fluorescence. B) Four representative rounds of our colocalization algorithm. Upper panels, pixel populations that contribute positively to the *r*×*R* coefficient; middle panels, pixel populations that contribute negatively to the *r*×*R* coefficient and lower panels, the remaining population of pixels. C) Graph bar representing the amount of pixels in the masks shown on B). As the algorithm proceeds with the detection and classification, the positive and negative populations increase, as the remaining population decreases.

Our algorithm proceeds by subsequently classifying pixels as colocalized or anti-colocalized in accord to the *r* and *R* level curves. A simple way of visualizing the practice of the algorithm is by combining the 2D histogram derived from the pair of images with the *r*×*R* level curves generated from the image statistic parameters (Fig. 1A). In this graph, the intensity of a given pixel in the green image is used as the *x*-coordinate and the intensity of the corresponding pixel in the red image as the *y*-coordinate. Thus, pixels that display fluorescence only in the green or red channel will lie on the *x* or *y* axis, respectively, pixels that display fluorescence in both channels will be placed along a diagonal, and pixels belonging to background signal will accumulate in the origin of the histogram. The intensity of each pixel in the 2D histogram (highlighted in jet colour map) represents the frequency of pixels that display those particular red and green values. The approach starts considering the totality of the pixels in both images, and subsequently classifies them as colocalized and anticolocalized in accord to *r*×*R* level curves. Indeed, in every round of classification, the algorithm groups those pixels that contribute greater than the 0.8× the maximum value or lower than 0.8× the minimum value of the product *r*×*R*. As the algorithm proceeds, the number of pixels in the remaining population decreases, while the colocalized and anti-colocalized populations increase (Fig. 1C). It continues until the step in which more than 1% of the pixels classified belong to the background, and then it selects the most appropriate colocalization mask out of all the generated masks (Fig. 1B and 1C, see Materials and Methods). The output of the algorithm is a colocalization mask which is highly similar to the true colocalization mask that encloses the pixels deliberately set as colocalized when we generated the images (compare Fig. 1B and 1A).

The rationale for determining the contribution of every pixels fluorescence intensity to the *r*×*R* map instead of using any of the coefficients by itself is as follows: the individual contribution of every pixel to the *r* can be negative or positive (Table 1, Fig. S2), which in general we attribute to colocalized and anti-colocalized pixels. Background pixels will contribute positively to this coefficient pushing the coefficient's numerator towards 1. However, the contribution of background pixels to the *R* is close (albeit positive) to 0 (Table 1). Thus, the product *r*×*R* map will bring all the correlated pixels of the background towards 0 while maintaining the higher values of the colocalized population of pixels, and thus it increases the dissimilar contribution of background and signal.

### New colocalization algorithm, validation in simulated images

**Figure 2:**
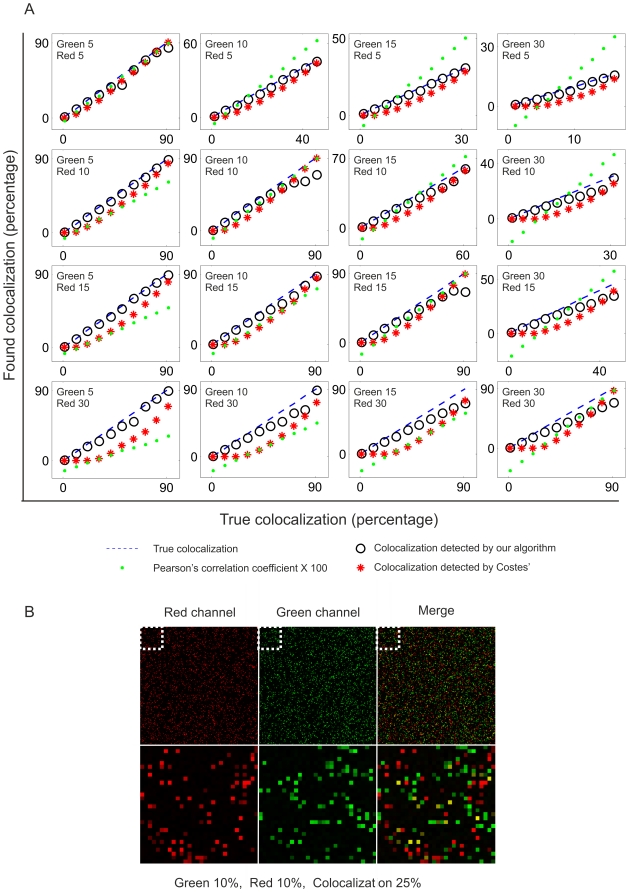
Performance of the new colocalization algorithm in simulated images. The accuracy of the algorithm was tested in a variety of simulated images with different colocalization extent. A) 160×160 pixel sized images were generated with different number of pixel-sized objects either located at random positions or in the same position (colocalized). Intensity distribution varied between 30 and 155 for both channels. Colocalized pixels/objects had identical non-zero intensities. Noise with intensities distributed uniformly between 10 and 30 was added to both channels. The density of each channel (percentage of the image covered by objects) varied and ranged from 5% to 30% for each green or red channel. Simulations covering the full range of possible colocalization were performed, from no colocalization to all green objects colocalized. The performance of our algorithm is plotted in black circles. Comparatively, it is plotted the performance of an algorithm written after Costes *et al*. [3] (red asterisks). Pearsońs correlation coefficient for each image pair is plotted multiplied by a factor of 100 (green dots). B) Example of a pair of images generated for this simulation.

We performed simulations covering different green and red object densities (this is, percentage of image area occupied by the objects) ranging from 5–30% and different amount of colocalization (from 0 to 100% of the green objects colocalized), and tested the accuracy of our algorithm and comparatively, an algorithm written after Costes *et al*., [3] to detect the existent colocalization. The new colocalization algorithm probed to be accurate and reliable for determining colocalization in most of the situations, even in those where the overall *r* was negative (Fig. 2A). However, Costes' algorithm was only accurate in images with low object density and could proceed only when the overall *r* was positive (Fig. 2A).

### New colocalization algorithm, validation in biological samples

**Figure 3:**
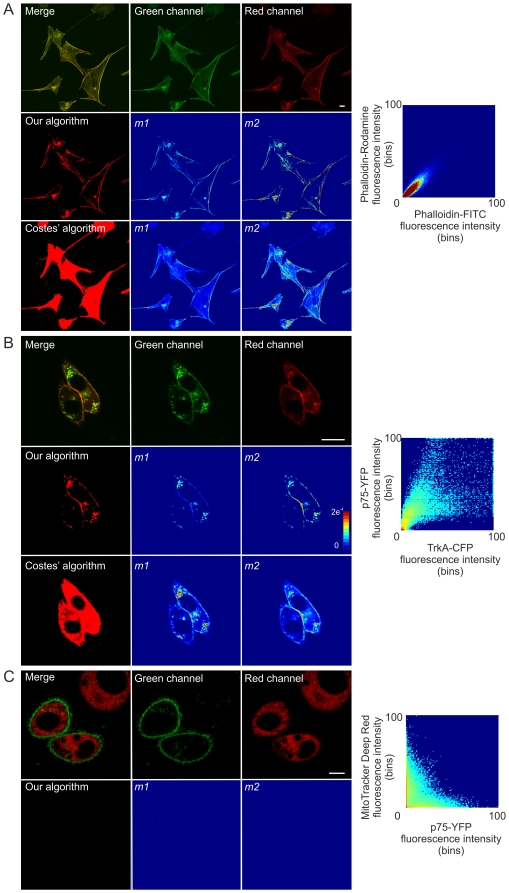
Performance of the new colocalization algorithm in biological samples. The accuracy of the algorithm was tested in a variety of biological images with a previously known colocalization extent. A, B and C are examples of whole, partial or no colocalization, respectively. Whole colocalization is achieved by labelling NIH cells with both Phalloidin-FITC and Phalloidin-Rhodamine. Partial colocalization is studied by transfection of TrkA-CFP and p75-YFP receptors into PC12 cells (Iacaruso *et al*., in preparation). Absence of colocalization is achieved by transfection of p75-YFP into HeLa cells and subsequent labelling with MitoTracker Deep Red. Green and red fluorescence channels and the merge of both channels are shown in every case. A mask enclosing the colocalized population of pixels detected with our algorithm is shown (middle left panel). Comparatively, the mask enclosing the colocalized population of pixels detected by the algorithm written after Costes *et al*. [3] (lower left panel). Additionally, the *m1* and *m2* maps generated with ours and comparatively, Costeś colocalization masks. On the right, two dimensional fluorescence histograms for each pair of images. Note the disposition of the pixels along the diagonal when colocalization is complete (A), or their segregation towards the *x* and *y* axis upon lack of colocalization (C). Bar, 10 µm. Jet colour bar, contribution of each pixel to the *m1* or *m2* coefficients (i.e., colocalized pixel intensity/whole image intensity). Bins, intervals of fluorescence intensity.

To determine the accuracy of our algorithm in biological samples, we compared different possible subcellular colocalization patterns (Fig. 3). A complete colocalization pattern was achieved by labeling actin cytoskeleton simultaneously with Phalloidin conjugated with FITC or Rodhamine (Fig. 3A). As a model for partial colocalization we studied NGF receptors fused to fluorescent proteins (TrkA-CFP and p75-YFP) that were characterized in our lab (Iacaruso *et al*., in preparation) or in previous literature [23] (Fig. 3B). Lack of colocalization was achieved by transfecting HeLa cells with p75-YFP and subsequently labeling with MitoTracker (Fig. 3C). For each pair of images, we constructed a 2D fluorescence intensity histogram, determined the colocalizing pixels with our algorithm and comparatively, with the algorithm written after Costes *et al*., [3] and constructed the *m1* and *m2* maps with the derived colocalizing masks (Fig. 3). Our algorithm successfully determined the actin filaments as colocalized in the model of complete colocalization, and some regions of the membrane and certain endosomes as colocalized in the model of partial colocalization, whereas Costeś algorithm defined the whole cell as colocalizing in both cases (Fig. 3). Our algorithm detected the lack of colocalization in Figure 3C whereas Costes' approach was unable to proceed due to the negative *r* value (Fig. 3).

### Time-lapse confocal fluorescence microscopy, image deconvolution and analysis of colocalization: validation for the study of kinases intracellular redistribution

We employed fluorescence confocal microscopy followed by image processing to address the intracellular redistribution of MEK, with particular interest in its traffic to mitochondria. We first validated the utilization of these techniques by evaluating the behavior of Akt1, which has been previously and extensively addressed [22]. Briefly, Akt is a serine/threonine kinase involved in cell proliferation, apoptosis, and glucose metabolism and is differentially activated by growth factors and oxidative stress by sequential phosphorylation of Ser473 by mTORC2 and Thr308 by PDK1. Phosphorylation of Thr308 in mitochondria determines Akt1 passage to nuclei and triggers genomic post-translational mechanisms for cell proliferation. At high H_2_O_2_, Akt1-PDK1 association is disrupted and thus, Akt1 phosphorylated at Ser473 accumulates in mitochondria in detriment to nuclear translocation; accordingly, the mutant Akt1 T308A is retained in mitochondria [22].

Here, we repeated the experiments of Antico-Arciuch and co-workers [22]. In these experiments, NIH/3T3 cells were transfected with wt Akt1-GFP or the respective mutants that lack one of the phosphorylation sites, Akt1 T308A-GFP and Akt1 S473A-GFP, and further stained with a specific mitochondrial marker, MitoTracker Deep Red, and analyzed by confocal microscopy. We determined the colocalization mask with our colocalization algorithm and measured GFP intensity inside this mask and normalized it to GFP intensity in the whole cell. In accord to the previous results, Akt1 T308A-GFP accumulated in mitochondria whereas Akt1 S473A-GFP localization in the organelle was scarce (Fig. S3A).

**Figure 4:**
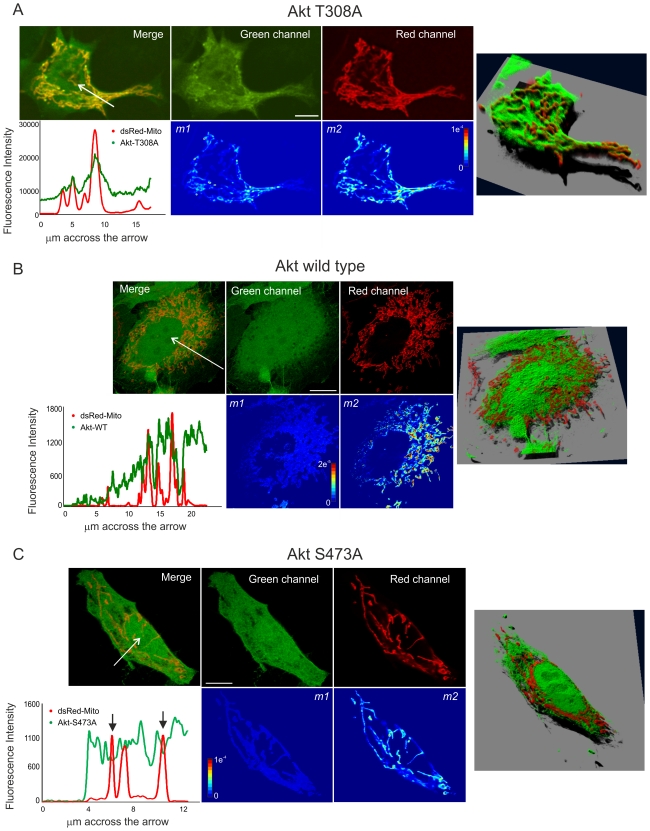
Image deconvolution: validation for the study of kinase translocation into mitochondria. HeLa cells co-transfected with mito-DsRed and either A) Akt1-T308A-GFP, B) Akt1-GFP, or C) Akt1-S473A-GFP were analysed by confocal microscopy and images were deconvolved with Huygens Deconvolution Software (Scientific Volume Imaging). A confocal plane of the deconvolved green and red channels is shown individually or merged (upper panels). Colocalization between the images was assessed by our colocalization algorithm and the *m1* and *m2* maps were generated utilizing the derived colocalization mask (lower panels). The fluorescence intensity profile across the arrow for both green and red channels is shown in the graph. Black arrows indicate the decrease in GFP inside mitochondrial regions. Bar, 10 µm. Jet colour bar, contribution of each pixel to the *m1* or *m2* coefficients (i.e., colocalized pixel intensity/whole image intensity). On the right, Simulated Fluorescence Process (SFP) volume. SFP volume is generated with an algorithm in which the data is taken as a distribution of fluorescent dye. By modelling a physical light/matter interaction process an image is computed showing the data as it would have appeared in reality when viewed under these conditions.

To improve the resolution of the images and more unequivocally determine the localization of Akt in mitochondria we employed image deconvolution. For these experiments, HeLa cells were transfected with Akt1 or its mutants and DsRed-mito. Images were specifically acquired and processed for image deconvolution. Deconvolved green and red fluorescence channels are shown in Figure 4. We determined the colocalization mask with our colozalization algorithm and constructed the *m1* and *m2* maps. Accordingly, Akt1 T308A-GFP accumulated in mitochondria as can be observed in the green fluorescence and merged images, in the *m1* map (yellow and red pixels) and in the fluorescence intensity profile in Figure 4A. Akt1 S437A-GFP localization to mitochondria was scarce as can be observed primarily in the dark *m1* map and in the fluorescence intensity profile (Fig. 4C). Thus, deconvolution analysis further corroborated the previously described Akt1 intracellular localization.

We then evaluated Akt redistribution upon stimulation with 50 µM H_2_O_2_, following the experiments of Antico-Arciuch and coworkers [22]. Transfected NIH cells were labeled with MitoTracker DeepRed and stimulated with H_2_O_2_ at the moment of image acquisition. GFP and MitoTracker fluorescence was followed every 1–2 min. We determined a colocalization mask with our colocalization algorithm for each time point and estimated the *m1* coefficient value by normalizing the GFP fluorescence intensity inside the mask per the GFP fluorescence in the whole cell. Akt1-GFP increased rapidly in mitochondria upon stimulation whereas the other variants did not display a major redistribution (Fig. S3B) as was previously reported.

**Figure 5:**
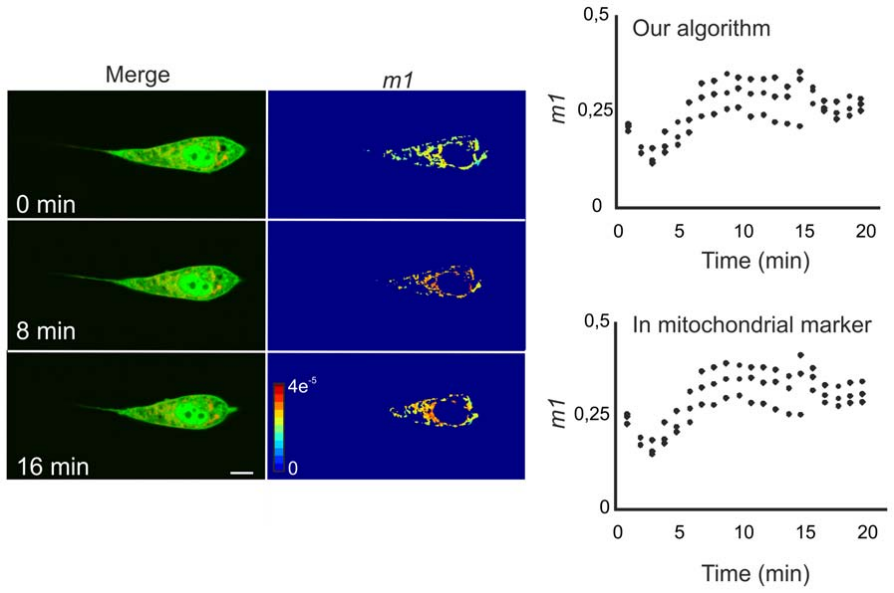
Performance of the new colocalization algorithm in the study of protein translocation kinetics. NIH/3T3 cells transfected with Akt1-GFP and stained with MitoTracker Deep Red were 24 h serum starved and subsequently stimulated with 10% fetal calf serum. Fluorescence intensity of both green (GFP) and red (Mitotracker) channels was followed for 20 min in a confocal microscope. The change in GFP fluorescence intensity after serum stimulation was analyzed in mitochondria by generating a colocalization mask with our colocalization algorithm or comparatively, generating a mask using MitoTracker fluorescence intensity [20], [22]. The graphs show the redistribution of Akt1-GFP in mitochondria assessed by computing the *m1* coefficient either with our algorithm or MitoTracker fluorescence generated masks for each of the 3 confocal planes analysed. A series of merged images and *m1* maps of representative time points after serum stimulation is shown on the left. Bar, 10 µm. Jet colour bar, contribution of each pixel to the *m1* coefficient (i.e., colocalized pixel intensity/whole image intensity).

To further corroborate the suitability of this analysis for kinase redistribution we compared it with a previously reported image processing and analysis of kinase redistribution. NIH cells were transfected and labelled as above and stimulated with FCS. Images were acquired every 1–2 min as above. We determined the change in GFP fluorescence intensity either in the colocalization mask generated with our algorithm or in a manually generated mask (see Materials and Methods) [20], [22] and normalized to GFP intensity in the whole cell. The redistribution retrieved by both methods was similar (Fig. 5); Akt1-GFP entered into mitochondria upon serum stimulation.

We concluded that these techniques are suitable for the study of kinase intracellular localization and redistribution.

### Presence and translocation of MEK1 into cellular and isolated mitochondria

**Figure 6:**
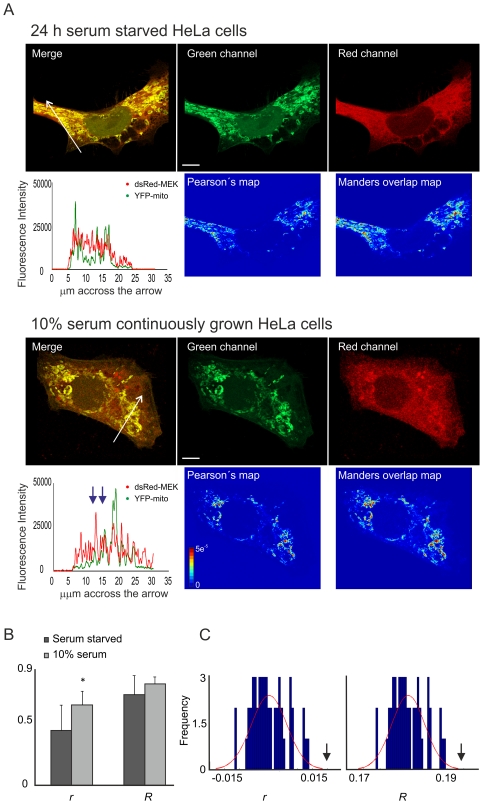
MEK1 is present in mitochondria of HeLa cells. A) HeLa cells transfected with mito-YFP and DsRed-MEK1 either 24 h serum starved or continuously grown in fetal calf serum were analysed by confocal microscopy and images were deconvolved with the Huygens Deconvolution Software (Scientific Volume Imaging). Deconvolved green and red channels of a confocal plane are shown individually or merged (upper panels). The Pearsońs correlation coefficient and Manders Overlap coefficient maps (lower panels) are also shown. The fluorescence intensity profile across the arrow for both green and red channels is shown in the graph. Blue arrows indicate an increase in MEK fluorescence intensity in mitochondrial areas. Bar, 10 µm. Jet colour bar, contribution of each pixel to the *m1* coefficient (i.e., colocalized pixel intensity/whole image intensity). B) Pearsońs correlation coefficient (*r*) and Manders overlap coefficient (*R*) were estimated for the image enclosed in the colocalization mask generated by our algorithm, for both serum starved and serum grown cells. *R* was significantly higher for cells continuously grown in serum, which suggests that MEK accumulates in mitochondria in this condition (*p* = 0.055, n = 6, Students *t* test). C) Significance of *r* and *R* was determined by comparison with those *r* and *R* values obtained when one of the images was repeatedly scrambled. The graph shows the empirical distribution of *r* and *R* values for independent (scrambled) images. Red line, *R* or *r* distribution adjusted to a normal fit. The *R* and *r* values obtained for the original pair of images is far beyond the probability distribution of random *r* or *R*, indicated by the black arrows.

In this work we demonstrated the presence of MEK1 in mitochondria of HeLa and MEF cells. In the first experiments, HeLa cells were transfected with DsRed-MEK1 and YFP-mito and images were acquired for deconvolution with or without prior serum starvation. The deconvolved images together with the *r* and *R* maps and an intensity profile are shown in Figure 6. We observed that MEK localization in mitochondria was augmented in non-starved cells (yellow and red pixels in *r* and *R* maps and coincident red and green fluorescence peaks in intensity profile). Furthermore, we computed the overall *r* and *R* inside the cell for both the starved and serum grown cells and observed that *r* was significantly higher in serum grown cells (Fig. 6B). We tested the significance of *r* and *R* in every case by assessing the empirical distribution of random coefficients, achieved by subsequently scrambling one of the images, and determined that the coefficients of the unscrambled -original- images were significant in both cases (Fig. 6C).

**Figure 7:**
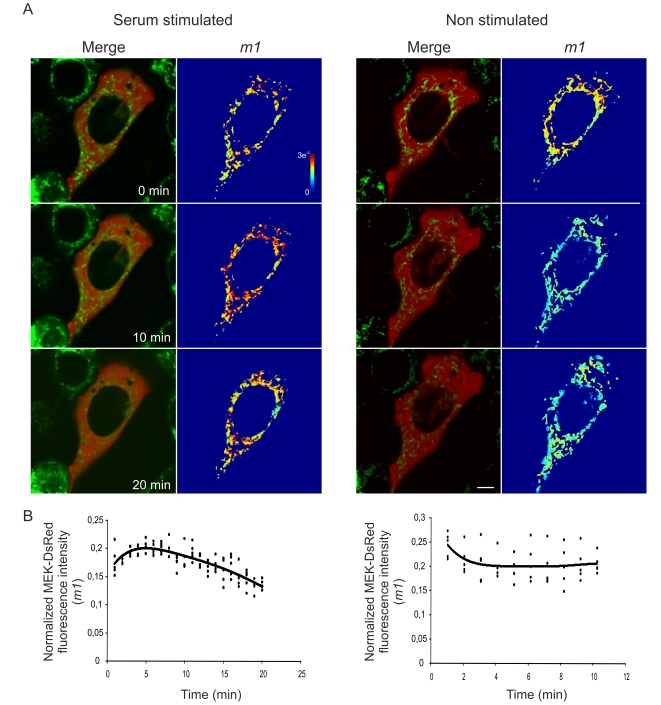
Kinetics of MEK1 translocation into mitochondria. A) HeLa cells transfected with DsRed-MEK1 and stained with MitoTracker Green were 24 h serum starved and subsequently stimulated with 10% fetal calf serum. Fluorescence intensity of both green (MitoTracker) and red (MEK) channels was followed for 20 min in a confocal microscope. The change in DsRed fluorescence intensity after serum stimulation was analyzed in mitochondria by generating a colocalization mask with our algorithm and subsequently generating the *m1* map and estimating *m1* coefficient value. A series of merged images and *m1* maps of representative time points after serum stimulation is shown on the left. For comparative purposes, MEK redistribution was evaluated in time prior to serum stimulation. A series of representative merged and *m1* images is shown on the right. Bar, 10 µm. Jet colour bar, contribution of each pixel to the *m1* coefficient (i.e., colocalized pixel intensity/whole image intensity). B) Changes in DsRed-MEK1 fluorescence intensity prior (right) or after (left) serum stimulation in mitochondria assessed by computing the *m1* coefficient using the colocalization mask generated with our algorithm for each of the 5 confocal planes analysed.

We then evaluated MEK redistribution upon FCS stimulation. For this, HeLa cells were transfected with DsRed-MEK1, labeled with MitoTracker green and 24 hs serum starved. At the moment of image acquisition, cells were stimulated with 10% FCS and imaged every 1–2 min. Analysis was performed by determining the colocalization mask with our colocalization algorithm and deriving the *m1* coefficient value as described above. Stimulation led to an early entrance of the kinase to the mitochondria followed by the exit of the organelle. Comparatively, we imaged and analyzed cells without FCS stimulation and observed no major redistribution (Fig. 7).

To confirm the localization of MEK in mitochondria, we isolated the organelle from HeLa cells, labeled them with MitoTracker DeepRed and subsequently fixed and immunostained them for MEK1/2 plus a secondary antibody conjugated to Cy3. Mitochondria were washed, mounted and analyzed by confocal microscopy. We observed the presence of endogenous MEK in purified non-contaminated mitochondria from HeLa cells (Fig. S4).

MEK localization to mitochondria was not particular of HeLa cells, as transfected DsRed-MEK1 was also present in mitochondria of MEF *erk1−/−* cells (Fig. S5), as well as its substrate ERK1 (Fig. S6). In both cases, the colocalization mask was determined with our colocalization algorithm and the *r* and *R* coefficients were calculated and were significantly higher than those obtained from independent (scrambled) images.

## Discussion

Here we present a new algorithm to determine the true colocalization in a pair of confocal fluorescence images without the bias of visual estimation. The algorithm is accurate and reliable for a variety of simulated and biological images and overcomes certain limitations of previous methods: i) it is functional for the whole range of object densities and percentage of colocalization in a pair of images; ii) it can proceed even when the overall *r* is zero or negative and, iii) it is accurate not only in ‘punctated’ images but also in situations where proteins are widely distributed throughout the cell. In a previous work, Costes and co-workers generated an algorithm that determined the colocalization based on the linear fit and the *r* of the pair of images [3]. These authors designed and algorithm capable to detect the smallest colocalization possible in images that contained few objects, i.e., few pixels with fluorescence intensity. Thus, the images employed contained a deliberately low object density (6–9%). The authors claimed that their algorithm was capable to detect pixels that contained colocalized signal that was not significantly above the mean intensity of the full image. They argued in those cases, it was impossible to identify colocalization by eye and thus, the algorithm had a sub-visual capacity to asses for colocalization. To accomplish with this challenge the algorithm sacrificed its precision in images where the colocalization between objects is high or asymmetric, or in those images where a high proportion of pixels contains fluorescence signal, i.e., high object density images (Fig 2) [6], [8]. Thus, care should be taken when applying this method in biological samples since these images tend to contain quite extensive object densities and variable degree of colocalization. Our colocalization algorithm was designed to contemplate this variability and was precise and consistent in a variety of simulated images including those where colocalization cannot be determined by eye (i.e., our algorithm has sub-visual capacity), and in the biological samples evaluated here as well as in other models. For example, in a former experiment Antico-Arciuch and co-workers [22] prepared a mixture of isolated mitoplasts and vesicles of NIH cells labelled with MitoTracker or an anti ATPase antibody, respectively, and additionally for phospho-Thr308 or phosphor-Ser473 Akt. The algorithm was employed to detect the colocalization of MitoTracker with ATPase and measure the fluorescence intensity of Akt in those vesicles that had ‘engulfed’ mitoplasts. This fluorescence intensity was compared to that found in vesicles that did not colocalize with MitoTracker (vesicles alone). The authors observed that P-Akt1 Ser473 label was predominantly in lone vesicles, whereas the highest P-Akt1 Thr308 fluorescence was detected in those vesicles that contained mitoplasts. This finding supported the notion that the plasma and mitochondrial membranes cooperate for the complete Akt activation in NIH cells and confirmed that Ser473 phosphorylation was a prerequisite for Thr308 phosphorylation to occur in mitoplasts [22]. In a different experiment, Iacaruso and co-workers followed the endocytosis kinetics of NGF receptors TrkA and p75 after NGF stimulation and confirmed by using our algorithm that TrkA endocyted rapidly while p75 remained in the cell membrane, and that there was a delayed appearance of colocalizing endosomes corresponding to the co-internalization of these receptors (Iacaruso *et al*., in preparation).

Costes' algorithm firstly calculates a least-squares fit for the 2D scattergram based on orthogonal regression and then progressively lowers intensity thresholds for both axes of the scatterplot and calculates the *r* between the pixels beneath the intensity thresholds of each channel until it reaches a pair of thresholds beneath which *r* is no longer positive. These thresholds are used to determine the cut-off for the calculation of *m1* and *m2*. The approach of Costes *et al*. therefore only considers positively correlated populations of pixels to be of interest -uncorrelated and anticorrelated pixels are ignored by their approach-, but also, due to its way of proceeding, it can only be applied to those images where the overall correlation is positive. This leaves out of the analysis a variety of images where the overall distribution is uncorrelated albeit still there is some minor but specific signal colocalization (Fig S2A) [27]. Our colocalization algorithm overcomes this inconvenient by independizing of the overall *r* value to proceed. Rather it contemplates the individual contribution of every pixel to both *r* and *R*.

**Figure 8:**
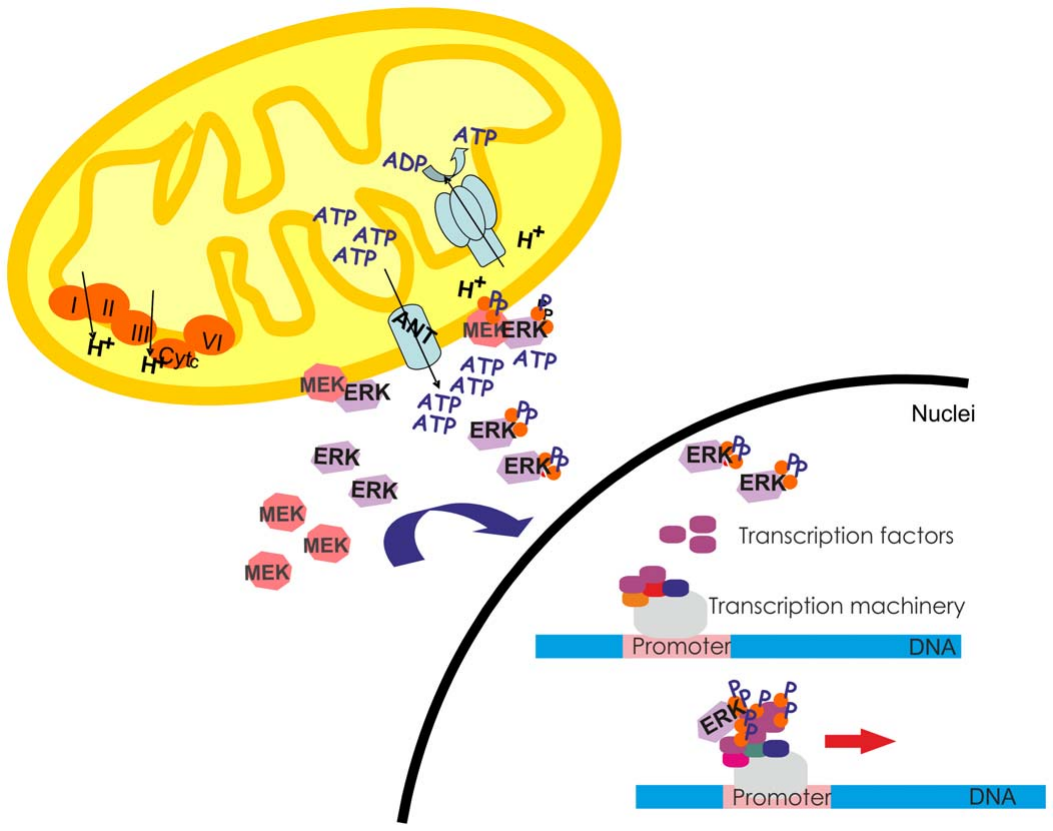
Intracellular redistribution of MEK and ERK. Scheme showing the new perspective of the redistribution of MEK and ERK inside the cell. As mitochondria are the source of ATP, MEK and ERK would go to the mitochondrial surroundings where phosphorylation might occur and afterwards ERK would translocate into the nuclei. There, ERK interacts with transcription factors and the transcription machinery to enhance RNA synthesis. The scheme was taken and modified from Galli *et al*. [20].

The exhaustive image analysis performed in the present work, as well as its statistical validation, support the notion of a real localization of recombinant DsRed-MEK1 in mitochondria of HeLa and MEF *erk1−/−* cells. Mitochondrial size ranges from 1–10 µm. Pixel size in these images was <140 nm for time-lapse microscopy images or <70 nm for images acquired for deconvolution. According to the optics, the minimal lateral and axial resolution gained with the microscope settings employed was approximately 150 nm and 400 nm, respectively. Thus, although mitochondria are above the diffraction limit, we incorporated image deconvolution to the analysis to improve the resolution and more unequivocally determine the presence of MEK in the organelle. We confirmed the presence of MEK in the organelle by immunostaining of isolated organelles with an anti MEK antibody. MEK localization in mitochondria augmented in proliferative conditions. Additionally, FCS stimulation of starved cells caused a rapid entrance of MEK into the organelle. In a previous work, we demonstrated that upon FCS stimulation of starved HeLa cells, hERK1-GFP entered the organelle with a maximum accumulation at around 5–10 min [20]. MEK accumulation in the organelle was faster, before 5 min of stimulation (Fig. 7). We have previously suggested that mitochondria are common sites for phosphorylation of proteins [19], [20], [22]. Here we propose that early transit of MEK to mitochondria would favor its phosphorylation, as c-Raf has also been reported in mitochondria [28], and in turn it would phosphorylate and activate ERK. Phosphorylated ERK would then shuttle into the nuclei and activate transcription (Fig. 8). Several experiments on permeabilized heart muscle fibers suggest the existence of diffusion restrictions grouping mitochondria and surrounding ATPases. The specific causes of these restrictions are not known, but intracellular structures are speculated to act as diffusion barriers. Indeed, Ramay y Vendelin [29] developed a three dimensional finite-element mathematical model in which the restriction barriers were composed by the sarcoplasmic reticulum, cytoskeleton proteins close to this reticulum and cytoskeleton proteins crowding that well adjusted the existent experimental data. This data supports our hypothesis in the sense that if mitochondria are source of ATP and the diffusion of this metabolite is restricted, it is expectable that kinases would reach the mitochondrial surroundings for activation prior to their translocation into the nuclei.

Both MEK and ERK have a role on mitochondrial metabolism regulation. For instance, Monick and co-workers [30] reported the presence of significant levels of MEK and ERK in mitochondria of alveolar macrophages and inhibition of ERK activity in these cells induced an early and profound depletion in cellular ATP, coincident with a loss of mitochondrial transmembrane potential. In concordance, Galmiche and co-workers [28] found c-Raf –which participates in MEK-ERK signalling cascade- in mitochondria of different cell lines and reported that c-Raf overexpression induced a change in mitochondrial morphology that rendered disaggregated organelles. This is consistent with our observations that overexpression of MEK or ERK induce mitochondrial fission as we did not observe the characteristic mitochondrial reticulum that we found in non-transfected cells (data not shown). Finally, we have recently shown evidence that suggests that mitochondrial ERK could induce mitochondrial genome expression [20]. Thus, the data from the present work in combination with early data from this and other labs propose a new frame for the study of kinase signalling pathways, in particular MEK-ERK cascade, that contemplates mitochondria as an obligatory station for activation, as well as a target for modulation of these kinases. The fact that kinase mutants that cannot be activated are retained in mitochondria in detriment of their nuclear translocation supports this notion [20], [22].

Together, we introduce a novel statistical approach to determine the colocalization in a pair of fluorescence images which is accurate in a surfeit of biological samples, ranging from small to large population of labelled molecules, lack to complete colocalization and even in those pair of images where the overall correlation is negative. This algorithm together with the construction of the more informative maps derived from the colocalization coefficients allowed us to directly observe in which parts inside the cell the colocalization was particularly relevant. Finally, all functionality of the methods here described was integrated into a friendly graphical user interface (GUI) environment for Matlab, designed for easy accessibility. This toolbox will be freely available for open source development (www.mathworks.com/matlabcentral/fileexchange/30665-villalta-et-al-s-colocalization-algorithm).

## Supporting Information

Figure S1 **New colocalization algorithm: end criterion.** A) Manders overlap coefficient is calculated inside the colocalized mask after each round of classification and is plotted vs. the round of classification. B) The area under this curve is calculated and normalized to the total area. The colocalization mask is the one attained when the area reaches ∼86%. In this simulation, the colocalization mask was determined in the iteration 21, with an area of 85.9% (dashed blue line in B and A). For this simulation, a pair of simulated images with ball objects was generated as in Figure 1. C) Colocalization mask determined by our algorithm. The number of circle objects was 17 for the red (D) and 14 for the green (E) images, with a final Pearson's correlation coefficient of 0.32.
(TIF)

Figure S2 **New colocalization algorithm, rationale.** The algorithm relies on the contribution of the individual pixels to the Pearsońs correlation and Manders overlap coefficients. Two images can be positively, negatively or not correlated in accord to the proportion of pixel pairs that contribute positively, negatively or null to the overall Pearsońs correlation coefficient A) Representative graphs showing how the distribution of pixels affect the overall coefficient value. B) Pearsońs correlation and Manders Overlap coefficient level curves represented in a three (left) or two (middle) dimensional histogram. The distribution was achieved by setting the maximum and mean fluorescence intensity to 255 and 75, respectively, for both the green and red channels, and computing the numerator of *r* or *R* for each possible pixel's fluorescence intensity combination (see Materials and Methods). Different contributions to the coefficients numerators highlighted in jet colour map. The masks over the 2D histograms (right) enclose pixel pairs that contribute positively to Pearsońs or Manderś numerator. Jet colour bar, values of the Pearsońs or Manderś numerator. Fluorescence intensity in bins.
(TIF)

Figure S3 **Presence and translocation of Akt1 and its phosphorylation mutants Akt-S473A and Akt-T308A into mitochondria.** NIH/3T3 cells transfected with Akt1-GFP, Akt-T308A-GFP or AktS-473A-GFP and stained with MitoTracker Deep Red were stimulated with 50 µM H_2_O_2_. Fluorescence intensity of both green (GFP) and red (Mitotracker) channels was followed for 20 min in a confocal microscope. A) GFP mean fluorescence intensity was quantified in the colocalization mask generated with our algorithm and normalized to whole cell mean GFP fluorescence before the stimulation. B) The change in GFP fluorescence intensity after H_2_O_2_ stimulation was analyzed in the colocalization mask generated with our algorithm and normalized to the total GFP intensity in the cell for all the Akt variants.
(TIF)

Figure S4 **MEK is present in isolated mitochondria of HeLa cells.** Isolated mitochondria from serum starved HeLa cells were labelled with MitoTracker Deep Red and further fixed and immuno-stained for MEK. Secondary antibody was conjugated to Cy3. An image of the individual and merged channels is shown (upper panels) together with a magnification (lower panels). Bar, 2.5 µm.
(TIF)

Figure S5
**Presence of MEK1 in mitochondria of MEF** ***erk1*** **−/− cells.** MEFs were transfected with DsRed-MEK1 and stained with MitoTracker Green. Images were acquired in a Zeiss LSM 510-meta confocal laser scanning microscope (Carl Zeiss, Thornwood, NY) with a 63×1.2 NA water immersion objective. Excitation and filters were as follows: MitoTracker Green, 488 nm excitation, emission BP 520
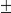
12 nm filter; RFP, 532 nm excitation, emission LP 585 filter. A) Green and red channels shown individually or merged. B) Colocalized pixels were determined with our colocalization algorithm and *m1* and *m2* maps were constructed with this mask. C) Significance of *r* and *R* was determined by comparison with those *r* and *R* values obtained when one of the images was repeatedly scrambled. The distribution of *r* and *R* values for independent (scrambled) images are shown. Red line, *R* or *r* distribution adjusted to a normal fit. *R* and *r* values obtained for the original images is far beyond the probability distribution of random *r* or *R* (black arrows). Middle bar graphs, Pearsońs and Manders coefficients. On the right Pearsońs and Manders maps. Bar, 2.5 µm. Jet colour bars, contribution of each pixel to the *m1*, *m2*, Pearson or Manders coefficient. These experiments were carried out in Dr. Jovin's laboratory.(TIF)

Figure S6
**Presence of ERK1 in mitochondria of MEF** ***erk1 −/−*** **cells.** MEFs were transfected with GFP-ERK1 and stained with MitoTracker CMXRos. Images were acquired in a Zeiss LSM 510-meta confocal laser scanning microscope (Carl Zeiss, Thornwood, NY) with a 63×1.2 NA water immersion objective. Excitation and filters were as follows: GFP, 488 nm excitation, emission BP 520
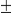
12 nm filter; MitoTracker, 532 nm excitation, emission LP 585 filter. A) Green and red channels shown individually or merged. B) Colocalized pixels were determined with our algorithm and *m1* and *m2* maps were constructed from this mask. C) Significance of *r* and *R* was determined by comparison with those *r* and *R* values obtained when one of the images was repeatedly scrambled. The distribution of *r* and *R* values for independent (scrambled) images are shown. Red line, *R* or *r* distribution adjusted to a normal fit. *R* and *r* values obtained for the original images is far beyond the probability distribution of random *r* or *R* (black arrows). Middle bar graphs, Pearsońs and Manders coefficients. On the right Pearsońs and Manders maps. Bar, 2.5 µm. Jet colour bars, contribution of each pixel to the *m1*, *m2*, Pearson or Manders coefficient. These experiments were carried out in Dr. Jovin's laboratory.(TIF)

Text S1 **New colocalization algorithm: end criterion.**
(DOCX)
