# Supplementary figures and images for: New Algorithm to Determine True Colocalization in Combination with Image Restoration and Time-Lapse Confocal Microscopy to Map Kinases in Mitochondria
Source: PLoS One. 2011 Apr 29;6(4):e19031. doi: 10.1371/journal.pone.0019031 (PMC3084741; doi:10.1371/journal.pone.0019031)

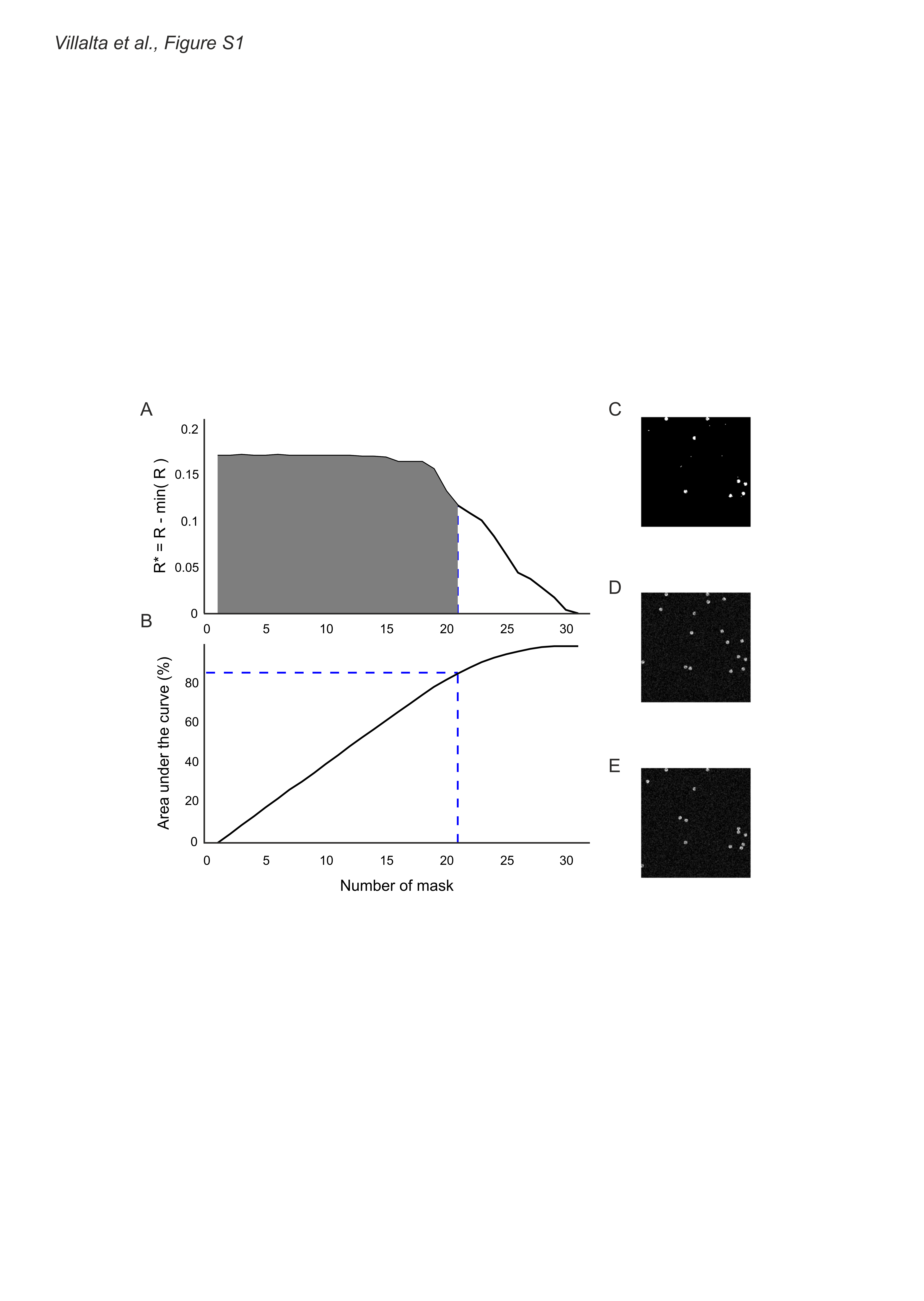

Supplement: Figure S1 — New colocalization algorithm: end criterion. A) Manders overlap coefficient is calculated inside the colocalized mask after each round of classification and is plotted vs. the round of classification. B) The area under this curve is calculated and normalized to the total area. The colocalization mask is the one attained when the area reaches ∼86%. In this simulation, the colocalization mask was determined in the iteration 21, with an area of 85.9% (dashed blue line in B and A). For this simulation, a pair of simulated images with ball objects was generated as in Figure 1. C) Colocalization mask determined by our algorithm. The number of circle objects was 17 for the red (D) and 14 for the green (E) images, with a final Pearson's correlation coefficient of 0.32. (TIF) [file pone.0019031.s001.tif]

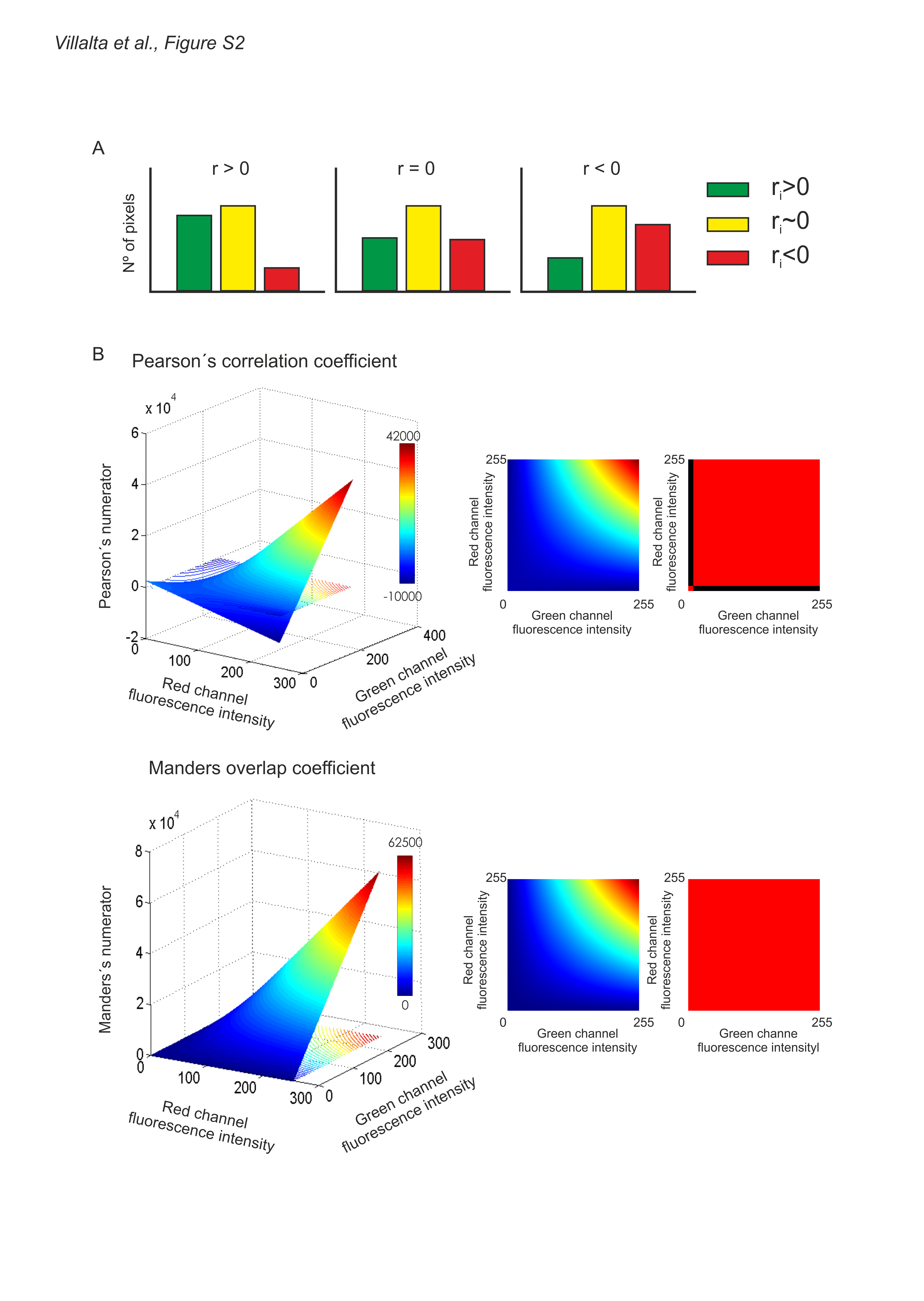

Supplement: Figure S2 — New colocalization algorithm, rationale. The algorithm relies on the contribution of the individual pixels to the Pearsońs correlation and Manders overlap coefficients. Two images can be positively, negatively or not correlated in accord to the proportion of pixel pairs that contribute positively, negatively or null to the overall Pearsońs correlation coefficient A) Representative graphs showing how the distribution of pixels affect the overall coefficient value. B) Pearsońs correlation and Manders Overlap coefficient level curves represented in a three (left) or two (middle) dimensional histogram. The distribution was achieved by setting the maximum and mean fluorescence intensity to 255 and 75, respectively, for both the green and red channels, and computing the numerator of r or R for each possible pixel's fluorescence intensity combination (see Materials and Methods). Different contributions to the coefficients numerators highlighted in jet colour map. The masks over the 2D histograms (right) enclose pixel pairs that contribute positively to Pearsońs or Manderś numerator. Jet colour bar, values of the Pearsońs or Manderś numerator. Fluorescence intensity in bins. (TIF) [file pone.0019031.s002.tif]

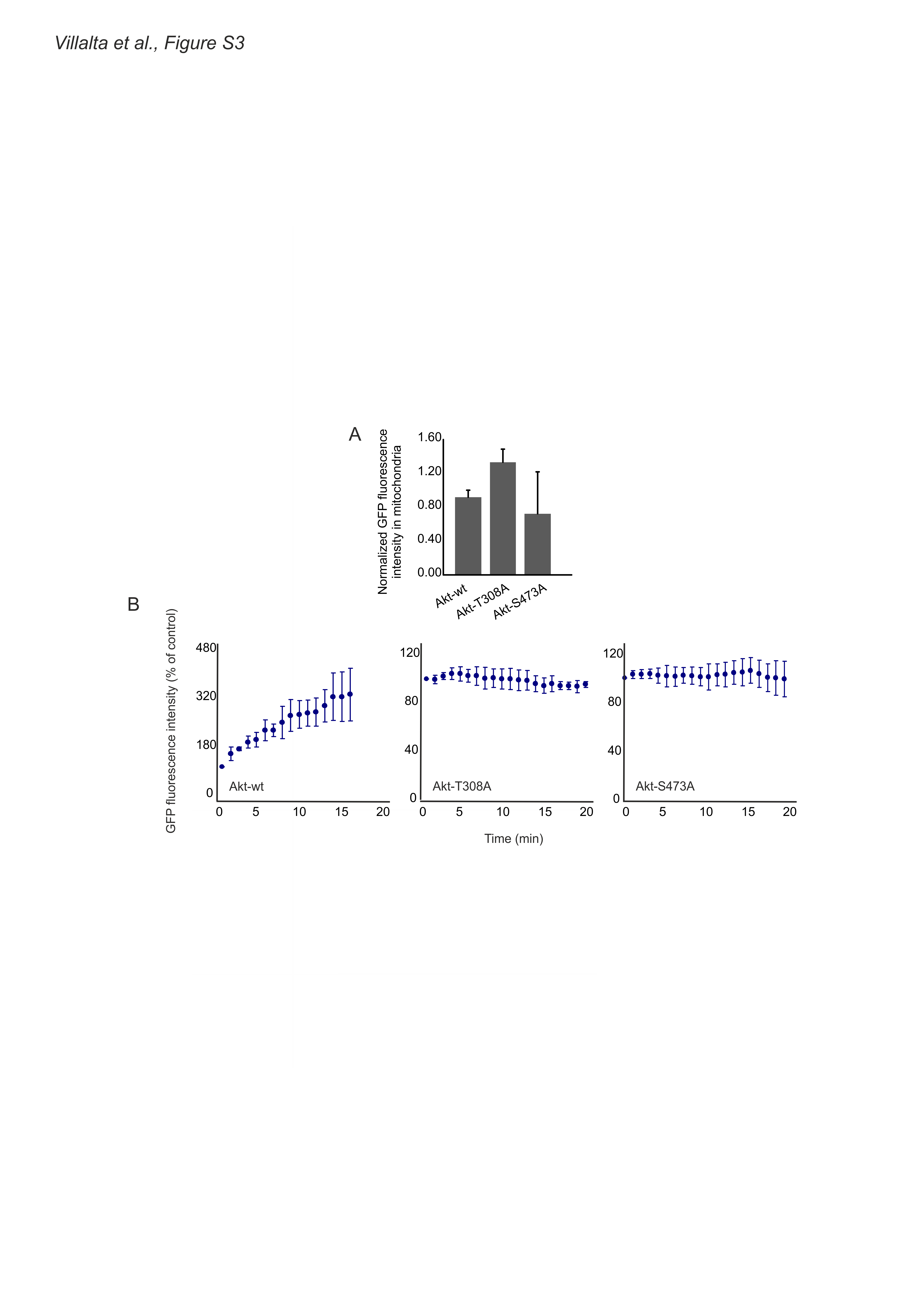

Supplement: Figure S3 — Presence and translocation of Akt1 and its phosphorylation mutants Akt-S473A and Akt-T308A into mitochondria. NIH/3T3 cells transfected with Akt1-GFP, Akt-T308A-GFP or AktS-473A-GFP and stained with MitoTracker Deep Red were stimulated with 50 µM H2O2. Fluorescence intensity of both green (GFP) and red (Mitotracker) channels was followed for 20 min in a confocal microscope. A) GFP mean fluorescence intensity was quantified in the colocalization mask generated with our algorithm and normalized to whole cell mean GFP fluorescence before the stimulation. B) The change in GFP fluorescence intensity after H2O2 stimulation was analyzed in the colocalization mask generated with our algorithm and normalized to the total GFP intensity in the cell for all the Akt variants. (TIF) [file pone.0019031.s003.tif]

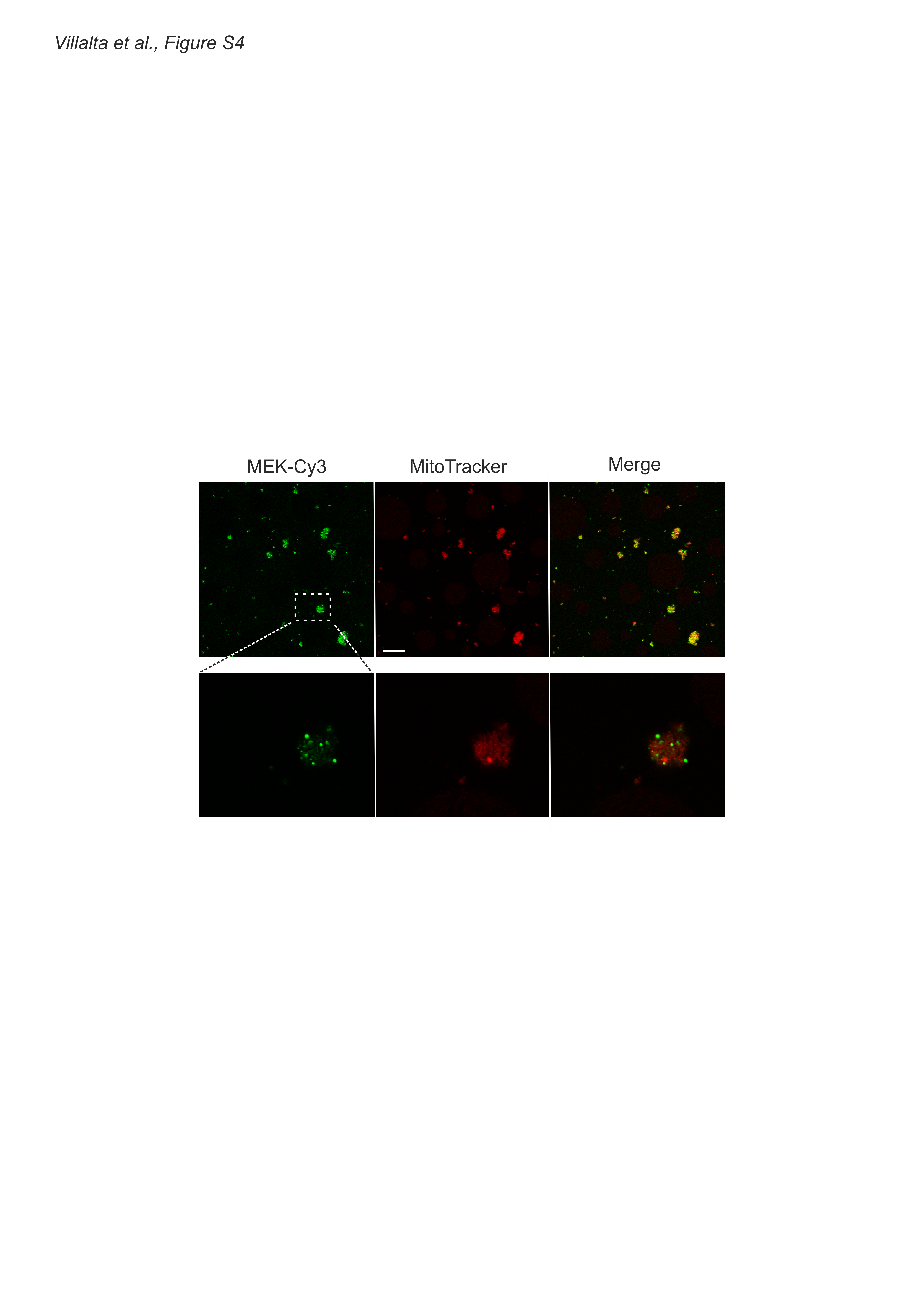

Supplement: Figure S4 — MEK is present in isolated mitochondria of HeLa cells. Isolated mitochondria from serum starved HeLa cells were labelled with MitoTracker Deep Red and further fixed and immuno-stained for MEK. Secondary antibody was conjugated to Cy3. An image of the individual and merged channels is shown (upper panels) together with a magnification (lower panels). Bar, 2.5 µm. (TIF) [file pone.0019031.s004.tif]

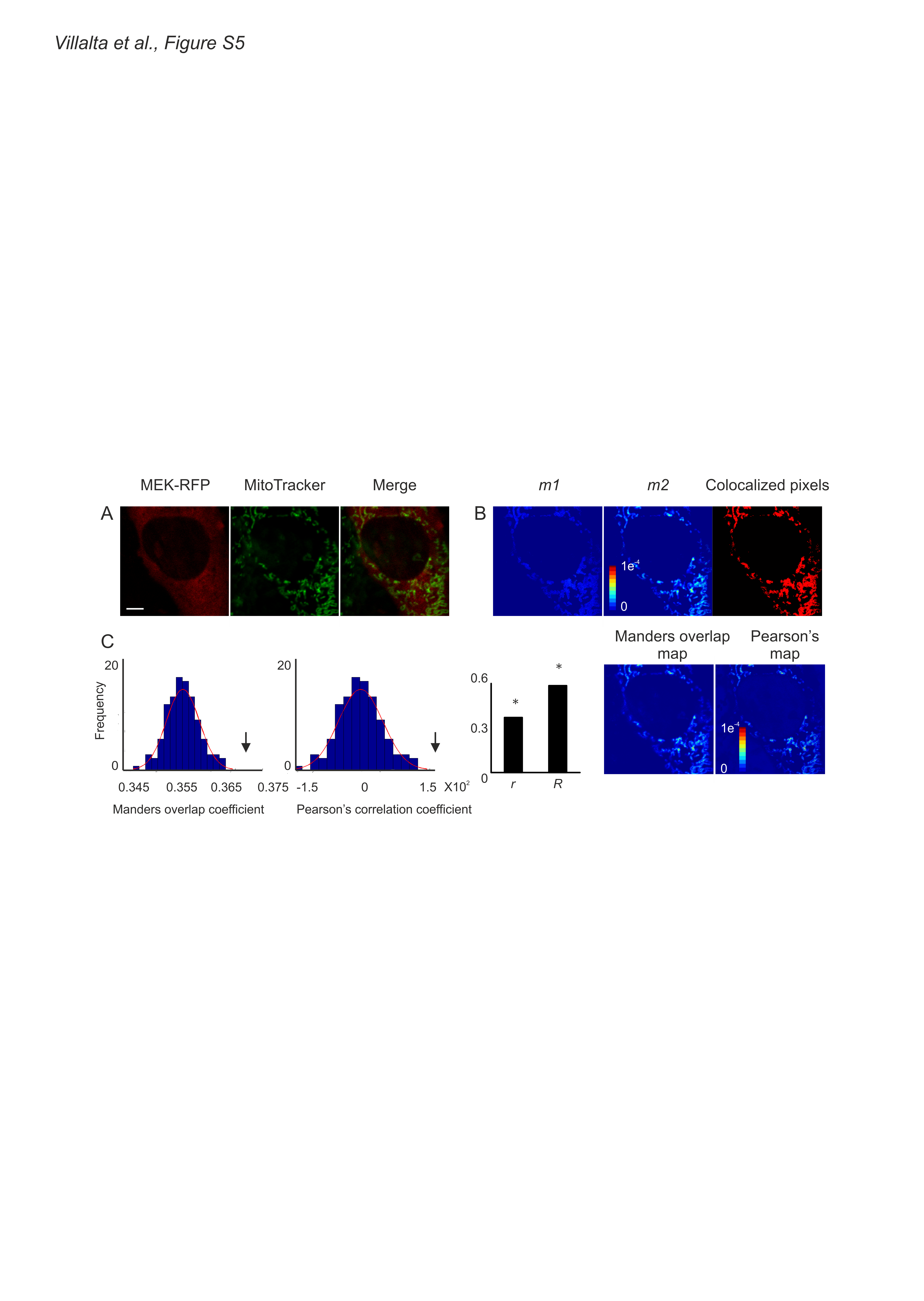

Supplement: Figure S5 — Presence of MEK1 in mitochondria of MEF erk1 −/− cells. MEFs were transfected with DsRed-MEK1 and stained with MitoTracker Green. Images were acquired in a Zeiss LSM 510-meta confocal laser scanning microscope (Carl Zeiss, Thornwood, NY) with a 63×1.2 NA water immersion objective. Excitation and filters were as follows: MitoTracker Green, 488 nm excitation, emission BP 52012 nm filter; RFP, 532 nm excitation, emission LP 585 filter. A) Green and red channels shown individually or merged. B) Colocalized pixels were determined with our colocalization algorithm and m1 and m2 maps were constructed with this mask. C) Significance of r and R was determined by comparison with those r and R values obtained when one of the images was repeatedly scrambled. The distribution of r and R values for independent (scrambled) images are shown. Red line, R or r distribution adjusted to a normal fit. R and r values obtained for the original images is far beyond the probability distribution of random r or R (black arrows). Middle bar graphs, Pearsońs and Manders coefficients. On the right Pearsońs and Manders maps. Bar, 2.5 µm. Jet colour bars, contribution of each pixel to the m1, m2, Pearson or Manders coefficient. These experiments were carried out in Dr. Jovin's laboratory. (TIF) [file pone.0019031.s005.tif]

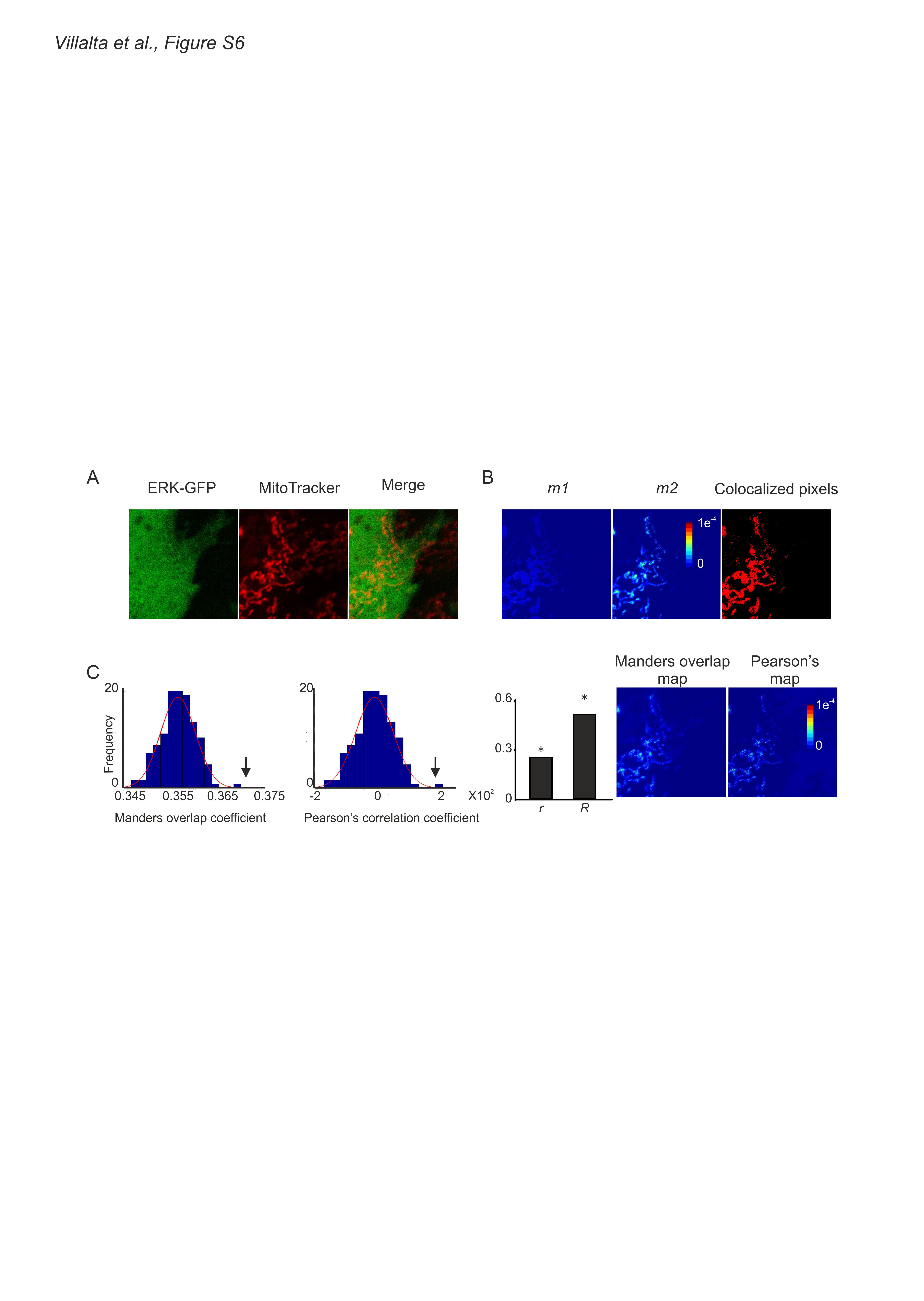

Supplement: Figure S6 — Presence of ERK1 in mitochondria of MEF erk1 −/− cells. MEFs were transfected with GFP-ERK1 and stained with MitoTracker CMXRos. Images were acquired in a Zeiss LSM 510-meta confocal laser scanning microscope (Carl Zeiss, Thornwood, NY) with a 63×1.2 NA water immersion objective. Excitation and filters were as follows: GFP, 488 nm excitation, emission BP 52012 nm filter; MitoTracker, 532 nm excitation, emission LP 585 filter. A) Green and red channels shown individually or merged. B) Colocalized pixels were determined with our algorithm and m1 and m2 maps were constructed from this mask. C) Significance of r and R was determined by comparison with those r and R values obtained when one of the images was repeatedly scrambled. The distribution of r and R values for independent (scrambled) images are shown. Red line, R or r distribution adjusted to a normal fit. R and r values obtained for the original images is far beyond the probability distribution of random r or R (black arrows). Middle bar graphs, Pearsońs and Manders coefficients. On the right Pearsońs and Manders maps. Bar, 2.5 µm. Jet colour bars, contribution of each pixel to the m1, m2, Pearson or Manders coefficient. These experiments were carried out in Dr. Jovin's laboratory. (TIF) [file pone.0019031.s006.tif]
