# Supplementary material for: New Algorithm to Determine True Colocalization in Combination with Image Restoration and Time-Lapse Confocal Microscopy to Map Kinases in Mitochondria
Source: PLoS One. 2011 Apr 29;6(4):e19031. doi: 10.1371/journal.pone.0019031 (PMC3084741; doi:10.1371/journal.pone.0019031)
Supplement: Text S1 — New colocalization algorithm: end criterion. (DOCX) [file pone.0019031.s007.docx]

New Algorithm to Determine True Colocalization in Combination with Image Restoration and Time-lapse Confocal Microscopy to Map Kinases in Mitochondria.

Jorge Ignacio Villalta^1*¥^, Soledad Galli^2,3*¶^, María Florencia Iacaruso^1ǂ^, Valeria Gabriela Antico Arciuch^4^^, Juan José Poderoso^3,4^, Elizabeth Andrea Jares-Erijman^2,3§^, Lía Isabel Pietrasanta^1,3§^.

Submission of revised manuscript PLoS One PONE-D-11-00713

**Supporting online material**

**Supporting information**

**New colocalization algorithm: end criterion**

The end criterion was experimentally determined. Briefly, we needed a way to analyze the behavior of the population of colocalized pixels after the subsequent rounds of classification and for this we tried the different coefficients alone or in combination (Pearson’s correlation coefficient, Manders overlap coefficient, or the product of both). We evaluated the change of these coefficients after each round of classification in a variety of simulated images that included single pixel objects or defined objects (circles) with a wide range of object densities and colocalization extent (15%-60% object density for each green and red channel for both pixel and circle objects, with colocalization extent ranging from 0 to 100% for pixel objects and 10-95% for circle objects, when the combination of object densities allowed). The cut-off was determined by contemplating the area under the curve of these functions. The best end criterion needed to satisfy two restraints: it had to achieve a colocalization mask highly similar to the true colocalization and it had to render the lowest amount of false positive pixels. The cut-off area was determined by averaging the areas attained for the whole set of images. We found that these restraints were best achieved by combining the evaluation of the Manders overlap coefficient and selecting the colocalization mask delivered by the round of classification in which the 86 % of the area was reached (Fig. S1). When using this end criterion, we were able to determine 83 ± 15% (n=196) of the existent colocalization of single pixel objects and 85 ± 9 % (n=150) for circle objects. The false positives generated were 0.2 ± 0.8% and 0.7 ± 0.9%, respectively.
